# Knockdown of GALNT1 suppresses malignant phenotype of hepatocellular carcinoma by suppressing EGFR signaling

**DOI:** 10.18632/oncotarget.3117

**Published:** 2015-02-04

**Authors:** Miao-Juei Huang, Rey-Heng Hu, Chih-Hsing Chou, Chia-Lang Hsu, Ya-Wen Liu, John Huang, Ji-Shiang Hung, I-Rue Lai, Hsueh-Fen Juan, Sung-Liang Yu, Yao-Ming Wu, Min-Chuan Huang

**Affiliations:** ^1^ Graduate Institute of Anatomy and Cell Biology, National Taiwan University College of Medicine, Taipei, Taiwan; ^2^ Department of Surgery, National Taiwan University Hospital, Taipei, Taiwan; ^3^ Department of Life Science, National Taiwan University, Taipei, Taiwan; ^4^ Institute of Molecular Medicine, National Taiwan University College of Medicine, Taipei, Taiwan; ^5^ Department of Clinical Laboratory Sciences and Medical Biotechnology, National Taiwan University College of Medicine, Taipei, Taiwan; ^6^ Research Center for Developmental Biology and Regenerative Medicine, National Taiwan University, Taipei, Taiwan

**Keywords:** GALNT1, GalNAc-transferase, O-glycosylation, epidermal growth factor receptor, receptor tyrosine kinase

## Abstract

O-glycosylation is a common protein modification. Aberrant O-glycosylation is associated with many cancers. GALNT1 is a GalNAc-transferase that initiates protein O-glycosylation. We found that GALNT1 is frequently up-regulated in hepatocellular carcinoma (HCC) and is associated with poor patient survival. Overexpression of GALNT1 increased and knockdown decreased HCC cell migration and invasion. Knockdown of GALNT1 inhibited EGF-induced migration and invasion. Knockdown of GALNT1 decreased EGFR activation and increased EGFR degradation, by decreasing EGFR O-glycosylation. This study demonstrates that down-regulation of GALNT1 is sufficient to suppress malignant phenotype of HCC cells by decreasing EGFR signaling. Thus, GALNT1 is a potential target in HCC.

## INTRODUCTION

Hepatocellular carcinoma (HCC) is a highly lethal cancer being the fifth most common cancer diagnosis and the third most frequent cause of cancer-related death worldwide [1, 2]. In Taiwan, HCC is the leading cause of cancer mortality with approximately 8000 new cases diagnosed and 7000 deaths occurring annually [3–5]. The mortality rate of HCC remains high as most patients present with unresectable advanced-stage disease, and recurrence following surgical resection remains a major problem as chemotherapy and radiation are of limited efficacy imposing a tremendous challenge in the management of HCC [6].

N-acetylgalactosaminyltransferase 1 (GALNT1) is a member of a large family of Golgi resident polypeptide N-acetylgalactosamine (GalNAc)-transferases (GALNT) that initiate mucin-type O-glycosylation by transfer of α-GalNAc from UDP-GalNAc to serine (Ser) or threonine (Thr) residues of proteins [7, 8]. The resultant short core region O-glycans, better known as cancer-associated Tn-antigens [9], are substrates of subsequent glycosidic chain extension, branching, and elongation by a repertoire of diverse glycosyltransferases [10]. Aberrant mucin-type O-glycosylation, both in structure and quantity, has been associated with cancers through altering the tumor cell-microenvironment interaction and influencing cell growth, survival, invasion, and metastasis, and lectin-receptor or immune system interaction [7]. Altered expressions of GALNT enzymes have been reported to associate with many cancers. GALNT2 down-regulation contributes to HCC cell malignant behaviors and enhances gastric cell proliferation and invasion [11, 12]. GALNT3 is frequently up-regulated in renal cell carcinoma (RCC) and independently predicts high-grade tumor and poor survival [13]; similarly GALNT3 is also up-regulated in high-grade serous epithelial ovarian cancer (EOC) and is correlated with shorter progression-free survival in advanced stage EOC [14]. Up-regulation of *GALNT14* was correlated with ovarian carcinogenesis [15]. *GALNT7* is frequently up-regulated in cervical cancer associated with cervical cancer cell proliferation, migration, and invasion [16]; yet down regulation of *GALNT1* and *GALNT7* is associated with enhanced melanoma cell migration, invasion and immunosuppression [17].

Very little is known about the function of GALNT1. Its expression is critical during early development of submandibular glands in mice through influencing the composition of extracellular matrix [18]. Knockout of GALNT1 in mice resulted in defective leukocyte recruitment [19]. O-glycosylation and Tn antigen expression have been reported in HCC [12, 20, 21]. *GALNT1* is the most highly expressed GALNT family genes in the liver [12]. However, no one has reported on the expression and function of GALNT1 in HCC. We therefore studied the roles of GALNT1 in HCC cellular behaviors and its clinical significance.

## RESULTS

### GALNT1 is frequently up-regulated in HCC and higher *GALNT1* expression levels are associated with poorer overall survival

**Figure 1:**
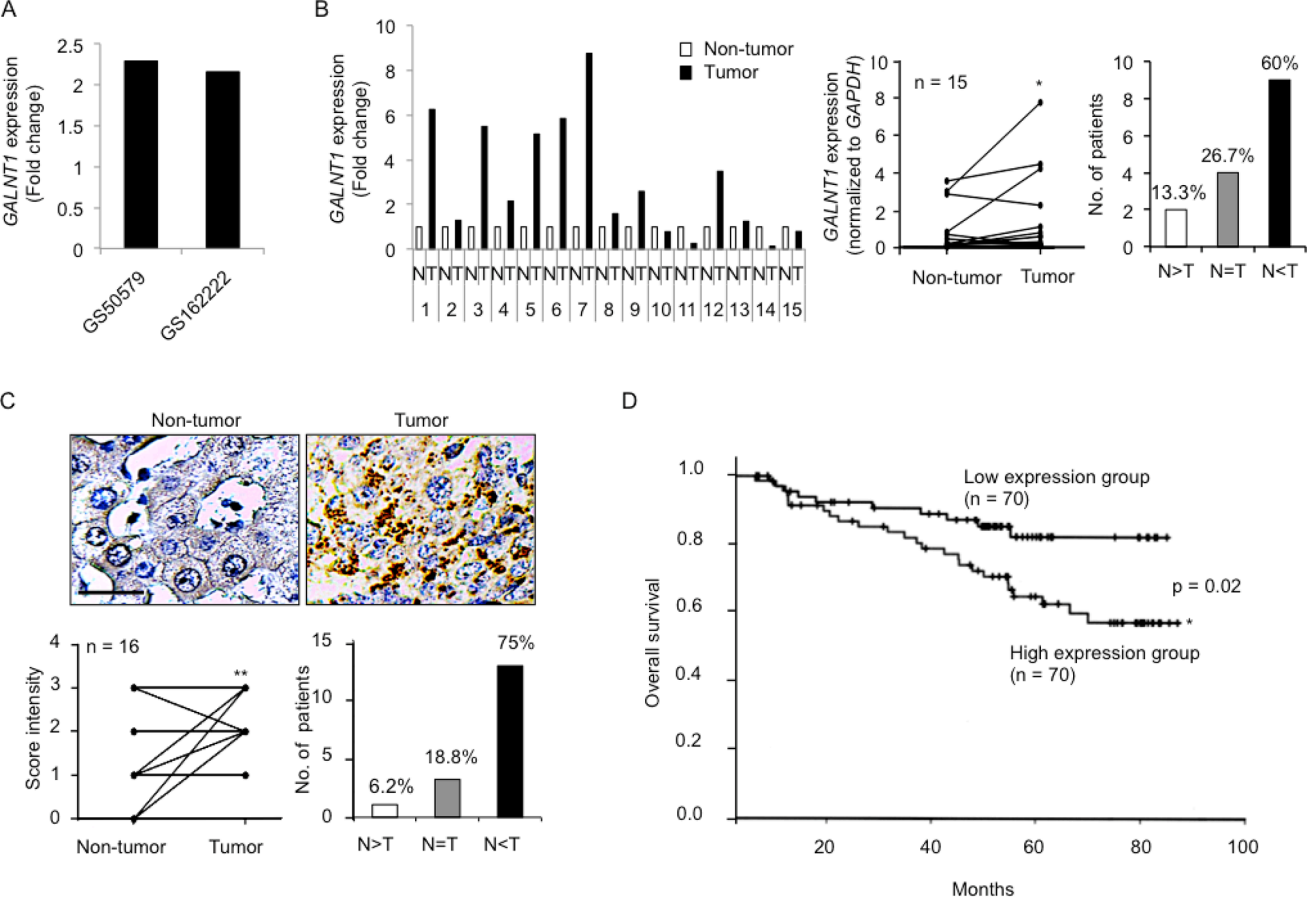
GALNT1 is frequently up-regulated in HCC and higher *GALNT1* expression levels are associated with poor overall survival **(A)** Resources analyzed from public databases (NextBio Research GS50579 and GSE62222) reveal that *GALNT1* expression is commonly increased in (1) hepatocellular carcinoma tumors compared with normal liver tissues (fold change: 2.29) and (2) hepatocellular carcinoma stage T3 compared with normal liver tissues (fold change: 2.16). *P* < 0.01. **(B)** Real-time RT-PCR quantification of *GALNT1* mRNA levels in 15 paired HCC tissues (normalized to *GAPDH* expression) and expressed as fold change in HCC tumor (T) compared with non-tumor tissues (N) (left panel). Sixty percent of the patients analyzed display higher *GALNT1* expression levels in HCC tumors compared with the adjacent non-tumor tissues, *p < 0.05. **(C)** Immunohistochemistry of GALNT1 of 16 paired HCC tissues. Representative images of non-tumor and tumor tissues are shown (upper panel), scale bar: 25 μm. GALNT1 intensity of HCC tumors was compared with their adjacent non-tumor parts (lower left) and 75% of the patients display increased GALNT1 expression levels in HCC tumors compared with non-tumor tissues, **p < 0.01. **(D)** *GALNT1* mRNA expression levels of 140 HCC tumors quantified by real-time RT-PCR in correlation with patient overall survival. HCC tumors expressing higher *GALNT1* levels (*n* = 70) are associated with deceased patient overall survival. *P < 0.05 is considered statistically significant.

To investigate the expression level of *GALNT1* mRNA in HCC, we first analyzed resources from the public database (NextBio Research). *GALNT1* mRNA expression levels are increased in HCC tumors (fold change: 2.29; GS50579) and in stage T3 HCC tumors (fold change: 2.16; GS50579) compared with normal liver tissues (Figure 1A). To confirm this finding, paired HCC tissues of 15 patients from the NTUH were collected for real-time reverse transcription polymerase chain reaction (RT-PCR) analysis (Figure 1B). The results reveal that *GALNT1* expression level is often increased in HCC tumors, *p* < 0.05, with 60% of the HCC patients exhibiting increased *GALNT1* expression levels in the tumors compared with the adjacent non-tumor tissues. Immunohistochemical staining of GALNT1 in 16 paired HCC tissues from the NTUH was performed and the staining intensity of tumor (T) and the adjacent non-tumor (N) tissues was scored from 0, +1, +2, and +3 for none, low, moderate, and high staining (Figure 1C). The immunohistochemistry (IHC) scores of HCC tumors were compared with the scores of the adjacent non-tumor tissues. The results further confirm that GALNT1 expression level is significantly increased in HCC tumors, *p* < 0.01, with 75% of the HCC patients exhibiting higher GALNT1 expression levels compared with the adjacent non-tumor tissues. To determine the correlation of GALNT1 expression with HCC clinicopathologic features we recruited 140 HCC tumors of patients from NTUH and analyzed for the *GALNT1* mRNA expression with real-time RT-PCR. Supplementary Table S1 displays the patients’ information. We found that HCC tumors exhibiting higher *GALNT1* expression levels are associated with poorer patient overall five-year survival (Figure 1D), *p* < 0.05. These findings show that GALNT1 is often overexpressed in HCC tumors and that higher *GALNT1* expression level is correlated with decreased HCC patient overall survival.

### GALNT1 expression regulates HCC cell malignant behaviors *in vitro*

**Figure 2:**
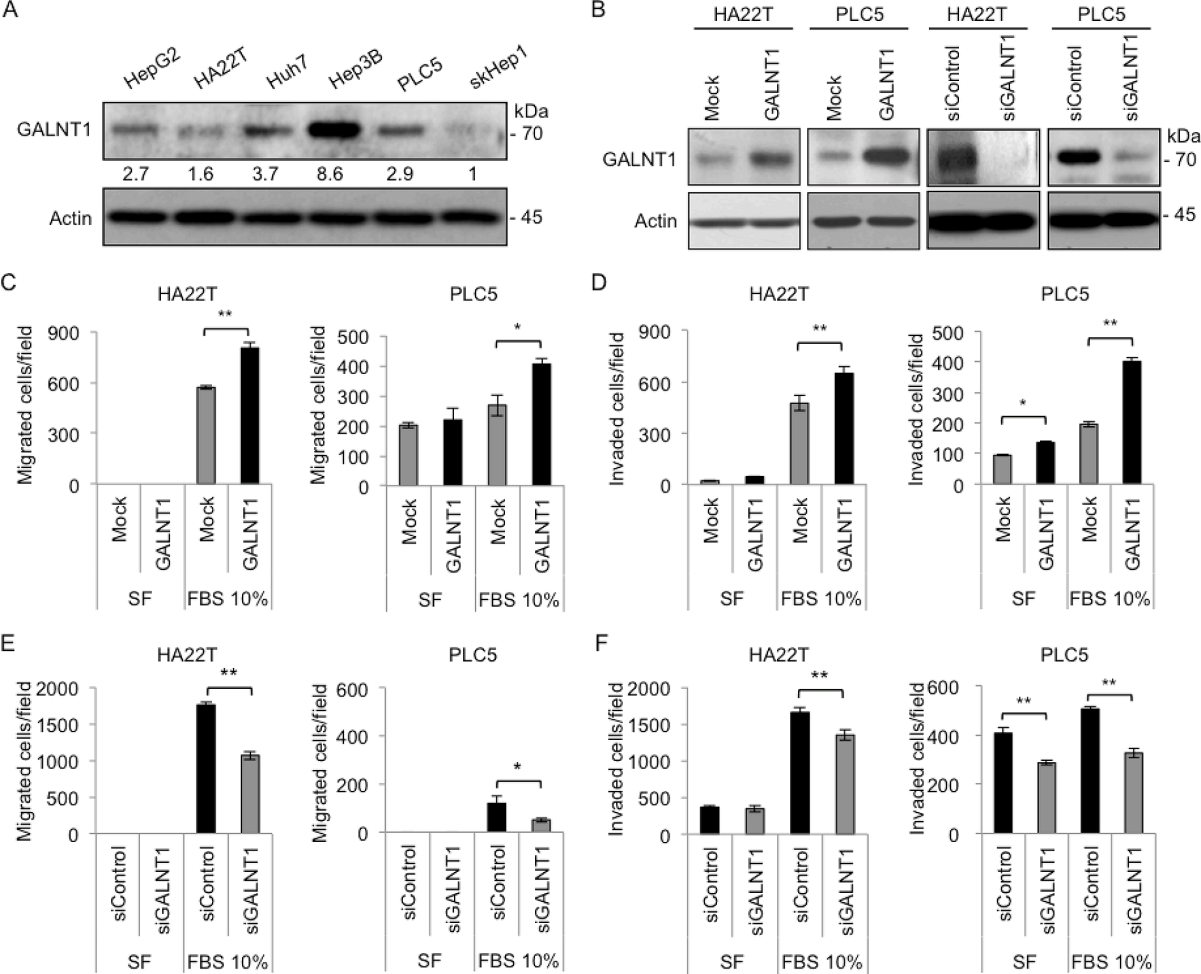
GALNT1 expression regulates HCC cell migration and invasion **(A)** Western blot of GALNT1 protein expression in multiple HCC cell lines. GALNT1 is differentially expressed in HepG2, HA22T, Huh7, Hep3B, PLC5, and skHep1 cells (upper panel) and expressed as relative expression intensity normalized to actin (lower panel) quantified by UN-SCAN-IT gel 6.1 software. **(B)** Western blots of GALNT1 overexpression by vector (Mock) or GALNT1/pcDNA3.1A (GALNT1) plasmids and knockdown by non-targeting (siControl) or GALNT1 siRNA (siGALNT1) in HA22T and PLC5 cells. **(C)** Overexpression of GALNT1 enhanced 10% FBS-induced HA22T (left) and PLC5 (right) cell migration. **(D)** Overexpression of GALNT1 enhanced HA22T (left) and PLC5 (right) cell invasion. **(E)** Knockdown of GALNT1 suppressed 10% FBS-induced HA22T (left) and PLC5 (right) cell migration. **(F)** Knockdown of GALNT1 suppressed HA22T (left) and PLC5 (right) cell invasion. Results obtained were analyzed by student's *t*-test and graphed as mean ± SD, **p < 0.01.

To investigate the effects of GALNT1 on HCC cell malignant behaviors, the *in vitro* cell viability, migration, and invasion assays were conducted. Western blot analysis reveals differential levels of GALNT1 expression in different HCC cell lines, namely, HepG2, HA22T, Huh7, Hep3B, PLC5, and skHep1 (Figure 2A). HA22T and PLC5 cells were selected for their intermediate GALNT1 expression levels to manipulate the expression of GALNT1 for further functional studies. Overexpression and knockdown of GALNT1 were achieved with GALNT1/pcDNA3.1A (GALNT1) plasmids and GALNT1 specific siRNA (siGALNT1), respectively, in HA22T and PLC5 cells and were confirmed by Western blotting (Figure 2B). The MTT assays showed no significant effects of GALNT1 on HCC cell viability (data not shown). However, using 10% FBS as chemoattractant, transwell migration and matrigel invasion assays demonstrate that overexpression of GALNT1 significantly enhanced HA22T and PLC5 cell migration (Figure 2C) and invasion (Figure 2D), *p* < 0.01. In contrast, knockdown of GALNT1 resulted in suppression of 10% FBS-induced HA22T and PLC5 cell migration (Figure 2E) and invasion (Figure 2F). These results suggest that overexpression of GALNT1 promotes whereas knockdown of GALNT1 inhibits HCC cell migration and invasion.

### GALNT1 expression regulates HCC cell malignant behaviors *in vivo*

**Figure 3:**
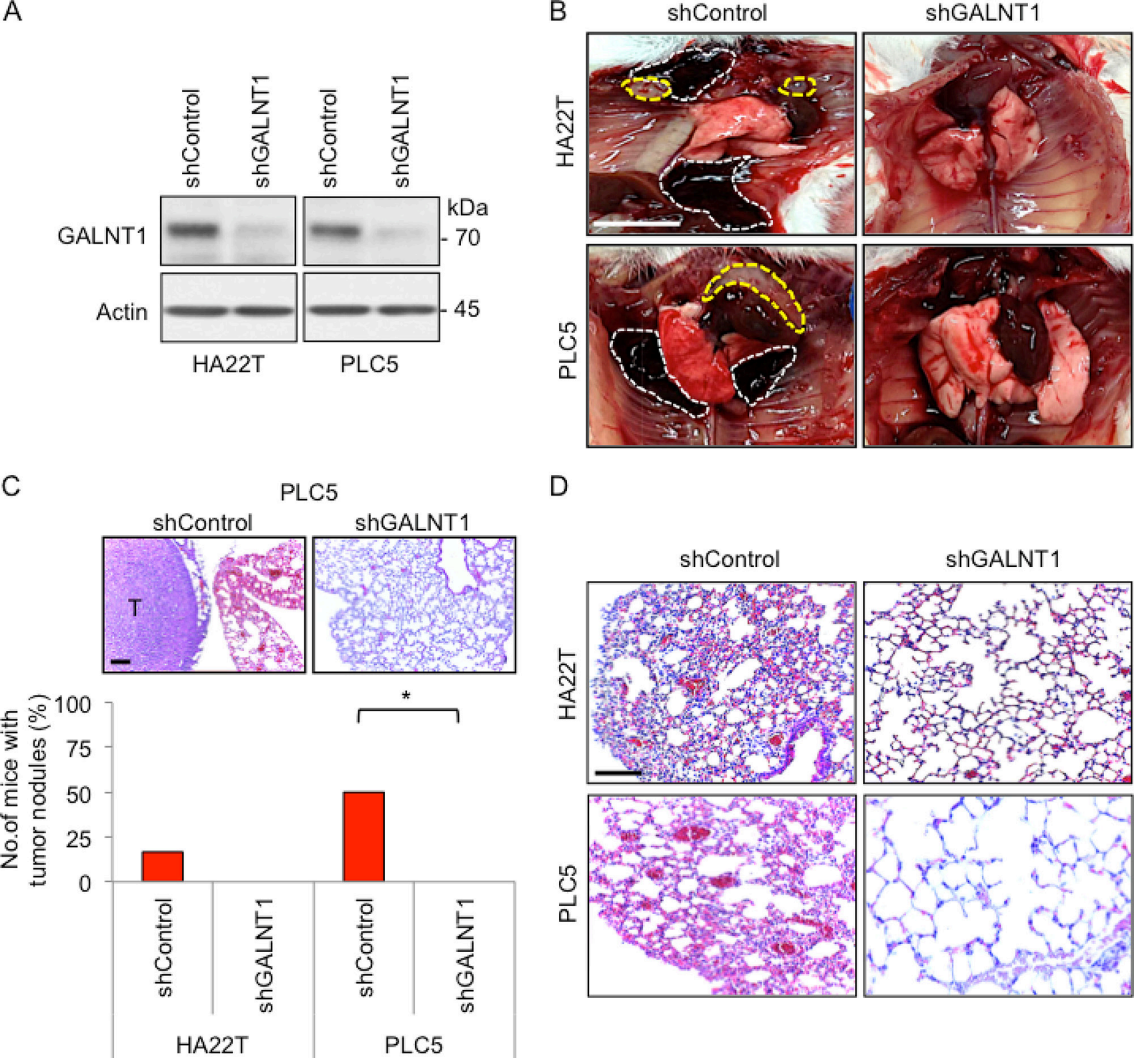
Knockdown of GALNT1 suppresses HCC cell metastasis *in vivo* **(A)** Stable knockdown of GALNT1 confirmed by Western blotting. **(B)** Stable transfectants of control or GALNT1 knockdown HA22T and PLC5 cells (5 × 10^6^) were injected into tail vein of NOD/SCID mice (*n* = 6 for each group). Metastatic tumor nodules (yellow discontinuous line) and hemorrhagic coagulant (white discontinuous line) were observed in the pleural cavity of the control group but not in the GALNT1 knockdown group. Scale bar: 1 cm. **(C)** Representative images of tumor nodules developed in PLC5 control group compared with GALNT1 knockdown group (upper panel), scale bar: 100 μm. Plot of the percentage of mice that developed metastatic tumor nodules. Twenty percent of the mice in HA22T control group while 50% of the mice in PLC5 control group showed pleuretic metastatic nodules. Chi-square analysis indicates knockdown of GALNT1 significantly suppressed metastatic tumor nodule development compared with control, *p < 0.05. **(D)** Knockdown of GALNT1 reduces HCC-induced lung injury. Representative histology of lung architecture of control and GALNT1 knockdown groups is shown. Compared with GALNT1 knockdown groups, lung tissues of HA22T and PLC5 control groups display alveolar wall thickening, perivascular and peribronchiolar distention, and vascular remodeling. Scale bar: 100 μm.

Since GALNT1 knockdown significantly affects HCC migration and invasion *in vitro*, we therefore investigated whether GALNT1 knockdown can suppress metastasis of HCC cells *in vivo* in NOD/SCID mice. Stable knockdown of GALNT1 in HA22T and PLC5 cells with shRNA was confirmed by Western blotting (Figure 3A). Control and GALNT1 knockdown HA22T and PLC5 cells were injected into the tail veins of NOD/SCID mice and sacrificed 60 days after injection. Metastatic tumor nodules and hemorrhagic coagulants were observed in the pleural cavity of the control mice (Figure 3B). Statistical results show that 20% of HA22T and 50% of PLC5 control mice developed tumor nodules (Figure 3C), *p* < 0.05. In contrast, neither metastatic nodule nor hemorrhagic coagulants were seen in GALNT1 knockdown groups of both HA22T and PLC5 cells. Knockdown of GALNT1 reduced HCC-induced lung injury development compared with control (Figure 3D). Interestingly, in contrast to the control, histology of the lung architecture of GALNT1 knockdown mice displays relatively normal lung architecture and alveolar integrity. However, despite no HCC tumor foci were observed, the lung tissues of mice bearing HA22T and PLC5 control tumors developed several structural modifications, including, alveolar wall thickening, perivascular and peribronchiolar distention, vascular remodeling, and increased macrophage infiltration (Figure 3D). These findings suggest that knockdown of GALNT1 suppresses HCC cell metastasis and HCC-induced lung injury *in vivo*.

### GALNT1 knockdown inhibits EGF-induced cell migration and invasion

**Figure 4:**
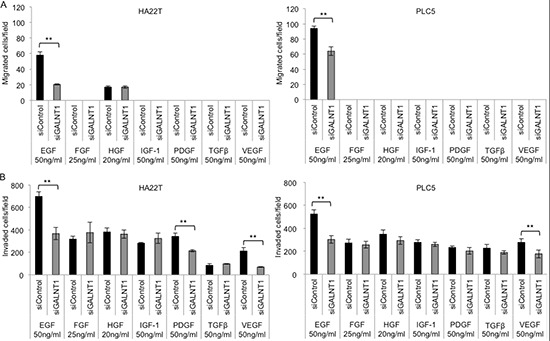
Knockdown of GALNT1 suppresses EGF-induced cell migration and invasion **(A)** GALNT1 knockdown suppressed EGF-induced cell migration in HA22T and PLC5 cells. **(B)** GALNT1 knockdown suppressed EGF-, PDGF- and VEGF-induced invasion in HA22T cells, and suppressed EGF- and VEGF-induced cell invasion in PLC5 cells. Results obtained were analyzed by student's *t*-test and graphed as mean ± SD, **p < 0.01.

To determine the target pathway of GALNT1 effecting HCC malignant phenotypes, a number of growth factors reportedly involve in HCC development [18–22] were tested using the transwell migration and matrigel invasion assays. Growth factors, including EGF 50 ng/ml, FGF 25 ng/ml, HGF 20 ng/ml, IGF-1 50 ng/ml, PDGF 50 ng/ml, TGFβ 50 ng/ml, and VEGF 50 ng/ml, in serum-free (SF) DMEM were used as chemoattractants. Knockdown of GALNT1 significantly suppressed EGF-induced migration (Figure 4A) and invasion (Figure 4B) in both HA22T and PLC5 cells, *p* < 0.01. In addition, GALNT1 knockdown also suppressed PDGF- and VEGF-induced invasion in HA22T cells and suppressed VEGF-induced invasion in PLC5 cells (Figure 4B). These results demonstrate that GALNT1 most significantly suppresses EGF-induced migration and invasion of HCC cells.

### GALNT1 knockdown reduces EGF-induced EGFR activation and facilitates EGFR degradation

**Figure 5:**
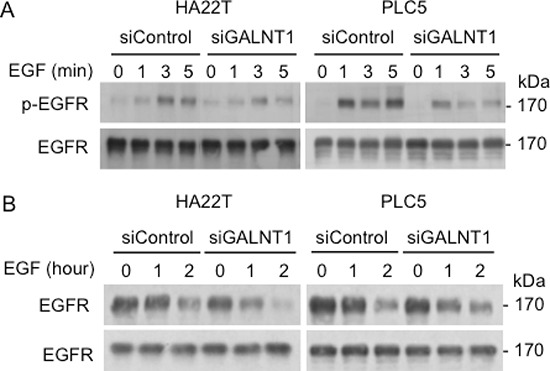
Knockdown of GALNT1 reduces EGF-induced EGFR activation and enhances EGFR degradation **(A)** GALNT1 knockdown suppressed EGF-induced EGFR phosphorylation. HA22T and PLC5 cells knockdown with non-targeting siRNA (siControl) and GALNT1 siRNA (siGALNT1) were serum starved overnight then treated with EGF 50 ng/ml for the indicated time points. Western blots of total cell lysates were immunoblotted with antibodies for p-Y1068 EGFR and total EGFR. A marked decrease in EGFR phosphorylation in both HA22T and PLC5 cells is evident at 3 min after EGF stimulation. **(B)** Knockdown of GALNT1 enhanced EGFR degradation. Control and GALNT1 knockdown HA22T and PLC5 cells were serum starved overnight, treated with cyclohexamide to inhibit protein synthesis, and then stimulated with EGF 50 ng/ml for the indicated time. Western blots depict decreased total EGFR levels from 1 h after EGF stimulation.

To investigate the mechanisms by which GALNT1 knockdown suppresses EGF-induced HCC cell migration and invasion, EGF-induced EGFR activation and degradation were analyzed. Western blots of total cell lysates immunoblotted with antibodies for EGFR p-Y1068 and total EGFR are shown (Figure 5A). GALNT1 knockdown suppressed EGF-induced EGFR activation where a decrease in p-Y1068 levels in both HA22T and PLC5 cells is clearly evident at 3 min. Next, we analyzed the effects of GALNT1 on EGFR degradation. Control and GALNT1 knockdown cells were serum starved overnight and then treated with EGF 50 ng/ml and/or cyclohexamide for 0, 1, and 2 hours. Western blot analysis shows that knockdown of GALNT1 enhanced EGFR degradation in HA22T and PLC5 cells (Figure 5B). Knockdown of GALNT1 did not affect *EGF* and *EGFR* mRNA expression levels in HA22T and PLC5 cells (Supplementary Figure S1). In addition, we also found that the expression level of EGFR is moderately correlated with GALNT1 expression levels in HCC tumors (r = 0.4894, *p* < 0.05) (Supplementary Figure S2). These results suggest that GALNT1 knockdown suppresses EGF-induced EGFR activation and enhances EGF-induced EGFR degradation in HCC cells.

### GALNT1 knockdown enhances EGFR co-localization with EEA1 and LAMP1

**Figure 6:**
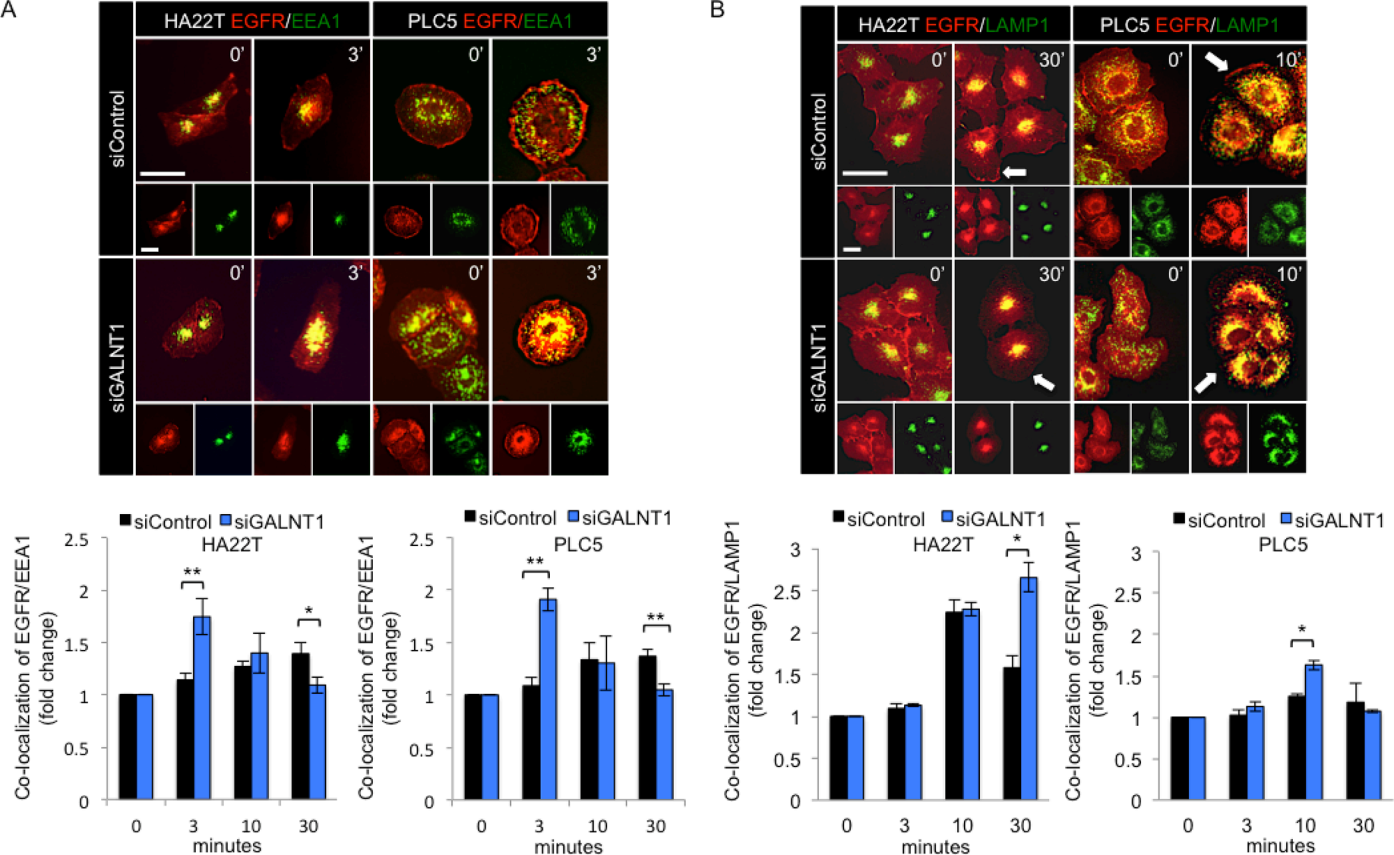
Knockdown of GALNT1 enhances EGFR co-localization with EEA1 and LAMP1 **(A)** Knockdown of GALNT1 enhanced EGFR co-localization with EEA1. Immunofluorescence staining of control (siControl) and GALNT1 (siGALNT1) knockdown HA22T and PLC5 cells with anti-EGFR (red) and anti-EEA1 (green) antibodies (upper panel). Cells were serum starved overnight and stimulated with EGF 10 ng/ml for 0, 3, 10, and 30 min and imaged by confocal microscopy. GALNT1 knockdown significantly enhanced EGFR co-localization with EEA1 at 3 min after EGF stimulation (lower panel). **(B)** Knockdown of GALNT1 enhanced EGFR co-localization with LAMP1. Control and GALNT1 knockdown HA22T and PLC5 cells were treated as above. Cells were immunostained for EGFR (red) and LAMP1 (green) after EGF stimulation (upper panel). Knockdown of GALNT1 significantly enhanced EGFR co-localization with LAMP1 at 30 min and 10 min after EGF stimulation in HA22T and PLC5 cells, respectively (lower panel). Representative merged images are shown with co-localization indicated in yellow. Nucleus was detected by DAPI (data not shown). Co-localization was quantified using Image J software. Results quantified are presented as fold change normalized to time 0. *P < 0.05, **p < 0.01; scale bar: 5 μm. Results were obtained from three independent experiments.

To investigate whether GALNT1 regulates EGFR trafficking, immunofluorescence staining of GALNT1 knockdown HA22T and PLC5 cells treated with EGF 10 ng/ml was performed to analyze for co-localization of EGFR (red) with early endosome antigen 1 (EEA1) (green) or lysosomal associated membrane protein 1 (LAMP1) (green) (Figure 6A and 6B, upper panels). EEA1 is an early endosome marker and LAMP1 is a lysosome marker. Quantification of co-localization (yellow) demonstrates that GALNT1 knockdown significantly enhanced EGFR co-localization with EEA1 at 3 min in both HA22T and PLC5 cells (Figure 6A, lower panel). Furthermore, GALNT1 knockdown significantly enhanced EGFR co-localization with LAMP1 at 30 min and 10 min in HA22T cells and PLC5 cells, respectively (Figure 6B, lower panel). Coinciding with these findings, GALNT1 knockdown triggered quicker onset of EGFR internalization with an enhanced EGFR co-localization with EEA1 and caused a marked decrease in cell surface EGFR most evident at 10 min compared with control (Supplementary Figure S3, arrows). Moreover, the cell surface EGFR continues to decrease while EGFR co-localization with LAMP1 increases in GALNT1 knockdown HA22T and PLC5 cells compared with control and results in an overall reduction of total EGFR at 30 min. These results suggest that GALNT1 knockdown accelerates EGF-induced EGFR internalization and co-localization with EEA1 and promotes receptor trafficking to the late endosome/lysosomal pathway leading to enhanced EGFR degradation.

### GALNT1 regulates EGFR O-glycosylation

**Figure 7:**
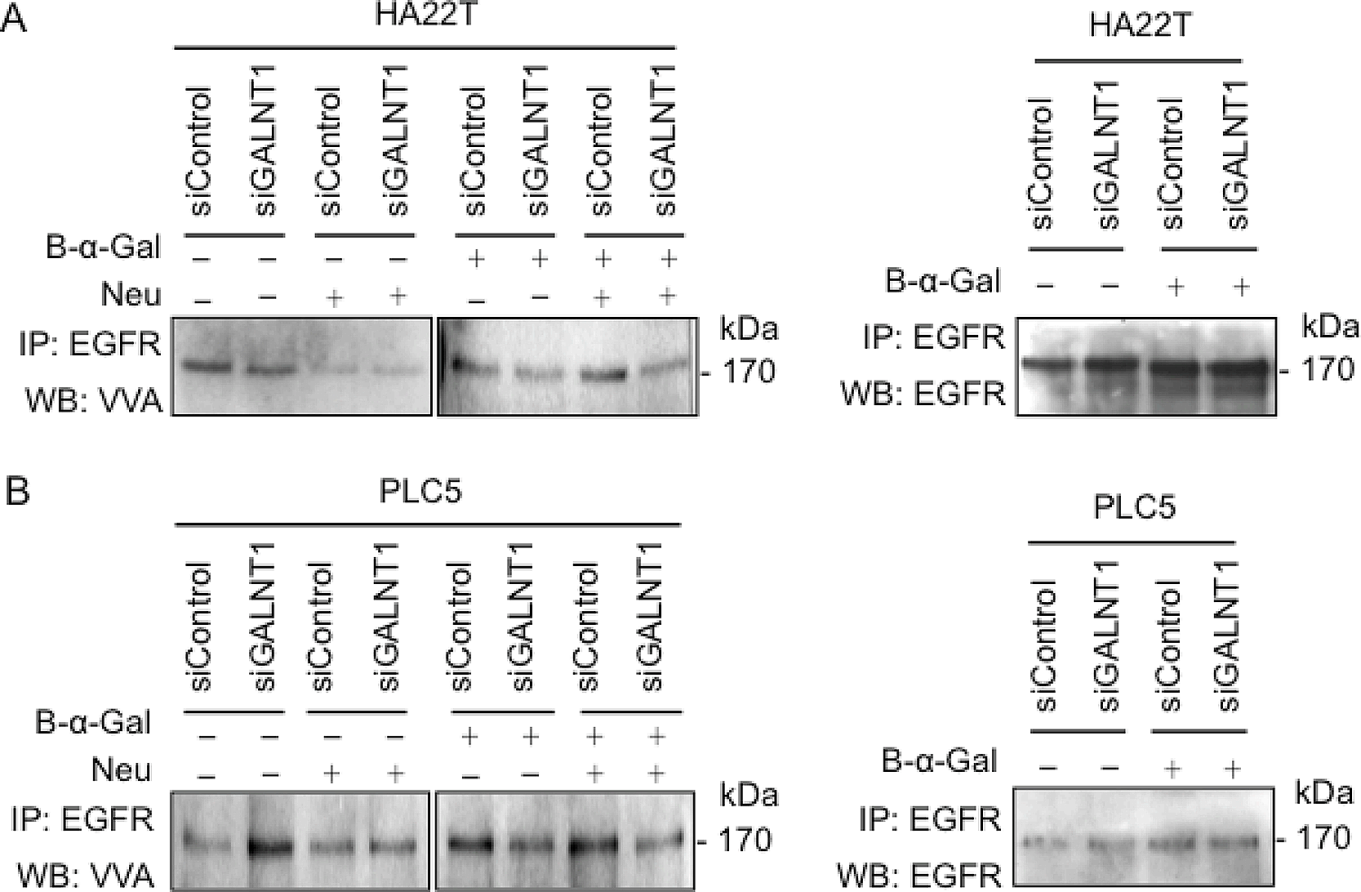
GALNT1 regulates EGFR O-glycosylation Effects of GALNT1 knockdown on VVA binding to EGFR in HA22T **(A)** and PLC5 **(B)** cells. Control and GALNT1 knockdown HA22T and PLC5 cells were treated with or without benzyl-α-GalNAc (B-α-Gal) to inhibit elongation and branching of O-glycans. Whole cell lysates were immunoprecipitated (IP) for EGFR and then incubated with or without neuraminidase (Neu) to unmask the effects of sialylation and Western blotted (WB) with biotin-conjugated VVA to distinguish Tn antigen expression. The effect of GALNT1 knockdown on reducing Tn antigen expression on EGFR was detected after treatment with benzyl-α-GalNAc, particularly after incubation with neuraminidase. Immunoprecipitated EGFR imunoblotted with anti-EGFR antibody serves as control.

To investigate whether GALNT1 affects the fate of EGFR through modifying EGFR O-glycans, VVA lectin was used to detect the level of Tn antigen (GalNAc-O-Ser/Thr) with change of GALNT1 expression. To minimize the effect of complex O-glycans and sialic acids on the lectin binding, benzyl-α-GalNAc treatment of cells and neuraminidase digestion of cell lysates were performed. EGFRs in control and GALNT1 knockdown HA22T and PLC5 cells were immunoprecipitated and blotted with biotin-conjugated VVA lectins (Figure 7A and 7B). In the absence of benzyl-α-GalNAc, we could not observe a decrease in Tn antigens on EGFRs in GALNT1 knockdown cells with or without neuraminidase treatment. We, therefore, treated the cells with benzyl-α-GalNAc to block further extension of Tn antigens. As expected, after treatment with benzyl-α-GalNAc, GALNT1 knockdown reduced VVA binding to EGFR O-glycans regardless of neuraminidase treatment. These results demonstrate that GALNT1 can modify O-glycans on EGFR in HCC cells.

### GALNT1 knockdown affects genes expression

**Figure 8:**
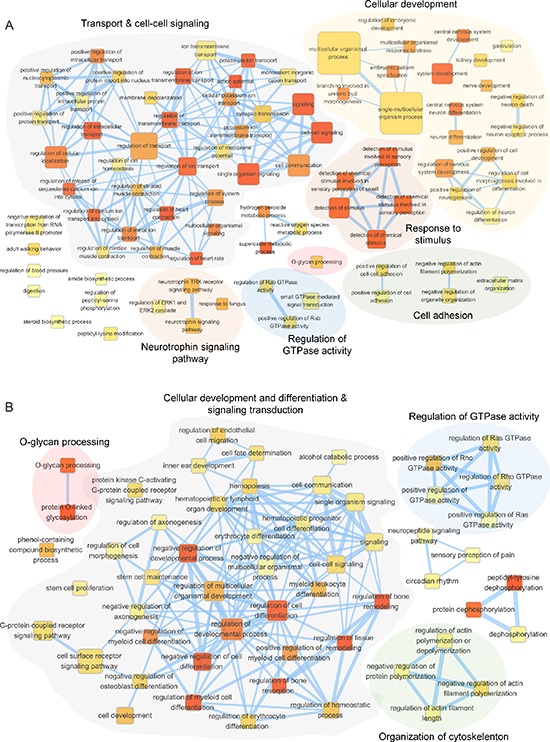
Functional maps of differentially expressed genes upon GALNT1 knockdown in HCC cells Enrichment results of *GALNT1* knockdown HA22T **(A)** and PLC5 **(B)** cells were mapped as networks. A node and each edge represent each enriched gene set (*p* < 0.05) and gene overlap score between nodes passing a threshold (threshold = 0.6), respectively. Node color encodes the enrichment *p*-value (red: low; yellow: high). The node size is proportional to the number of genes belonging to the corresponding gene set. The edge thickness is proportional to the overlap score. Groups of functionally related gene sets are highlighted in colors and as labeled.

**Figure 9:**
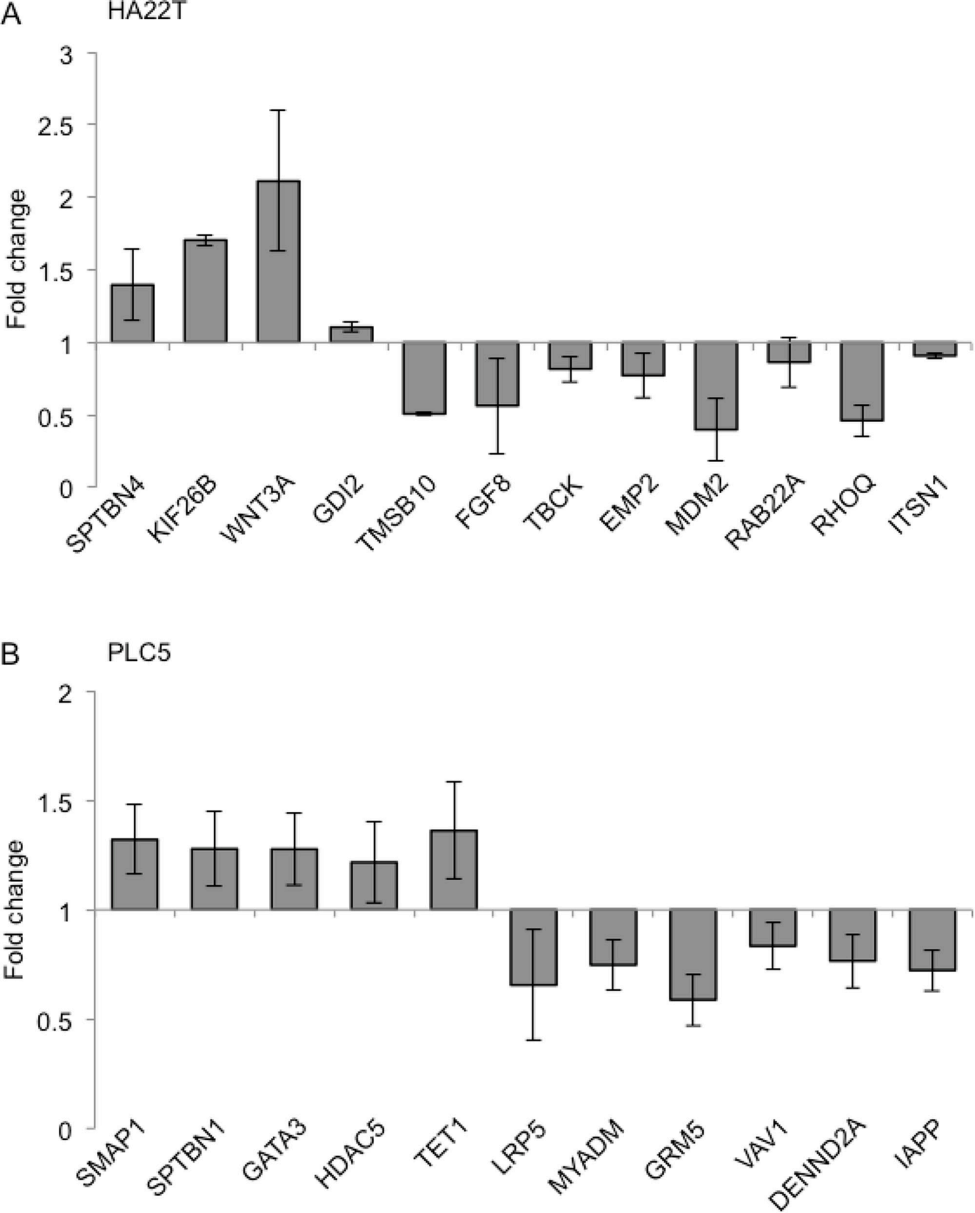
Quantitative RT-PCR validation of microarray results Differential expressions (fold change) of selected genes upon *GALNT1* knockdown in HA22T **(A)** and PLC5 **(B)** cells compared with control. Values greater than 1 represent gene up-regulation and values less than 1 represent gene down regulation. Results were analyzed from three independent *GALNT1* knockdown HA22T and PLC5 cells and their corresponding control and are presented as mean ± SD.

To better understand the molecular mechanisms delineating the role of *GALNT1* gene expression in HCC tumorigenesis, we evaluated the global gene expression changes in control and *GALNT1* knockdown HA22T and PLC5 cells. All microarray experiments were performed in triplicates where three hybridizations were conducted for each *GALNT1* knockdown cells against the corresponding control. Functional enrichment and network analysis reveal that *GALNT1* knockdown in HA22T cells leads to differential gene expression in pathways, including transport and cell-cell signaling, cellular development, response to stimuli, cell adhesion, regulation of GTPase activity, and neurotrophin signalling pathway (Figure 8A). *GALNT1* knockdown in PLC5 cells leads to differential gene expression pathways, including cellular development and differentiation, signal transduction, regulation of GTPase activity, and organization of cytoskeleton (Figure 8B). The differential expressions (≥ 1.5 fold change, *p* < 0.05) of functionally related groups of genes are provided in Supplementary Table S2. Genes selected for real-time RT-PCR validations are shown in Figure 9A and 9B. In HA22T cells, genes related to cell adhesion (WNT3 and KIF26B) were up-regulated; and genes regulating intracellular protein transport (MDM2 and RHOQ) were down-regulated. In PLC5 cells, genes regulating actin filament polymerization (SPTBN1) and RasGTPase activity (SMAP1) were up-regulated; and genes regulating cell surface receptor signaling (LRP5, GRM5, and IAPP) and RasGTPase activity (VAV1 and DENND2A) were down-regulated.

## DISCUSSION

GALNT1 plays important roles in the biosynthesis of O-glycans. Aberrantly expressed O-glycans have been reported in many cancers; however, the roles of GALNT1 in cancers remain largely unclear. In this study, we found that GALNT1 expression level is commonly up-regulated in HCC; and higher *GALNT1* level is associated with poor HCC patient survival. We discovered that GALNT1 overexpression enhances HCC cell migration and invasion. In contrast, knockdown of GALNT1 suppresses HCC malignant phenotypes *in vitro* and *in vivo*. Mechanistic investigation suggests that GALNT1 modifies EGFR O-glycosylation, thereby, regulating EGFR phosphorylation, internalization and degradation, which in turn modulates HCC cell malignant behaviors. This study is the first to report that GALNT1 plays important roles in HCC malignant behaviors.

Here, we show that GALNT1 can modify EGFR O-glycosylation in HCC cells. Unexpectedly, in the absence of benzyl-α-GalNAc treatment, GALNT1 knockdown resulted in increase of VVA binding to Tn antigens on EGFR. To explain this phenomenon we postulate that GALNT1 may be a GalNAc-transferase in HCC that initiates mucin-type O-glycosylation designated for further complex carbohydrates biosynthesis. Thus, knockdown of GALNT1 expression reduces complex O-glycans formation, but results in increased exposure of Tn antigens that are otherwise masked by steric hindrance. Indeed, when cultured in presence of benzyl-α-GalNAc to block O-glycan extension and branching, GALNT1 knockdown in both HA22T and PLC5 cells display reduced levels of Tn antigen on EGFR compared with control, regardless of the removal of sialic acids. We therefore conclude that GALNT1 is able to add GalNAc to EGFR in HCC cells.

EGF and EGF receptor activation have been shown to be involved in the development and progression of many cancers. Transgenic mice with liver-specific overexpression of EGF lead to rapid development of HCC [23, 24]. EGF-induced EGFR signaling regulated HCC proliferation, metastasis and production of inflammatory cytokine [25]. EGFR phosphorylation and protein levels are involved in EGFR signaling at very early and later stage, respectively. EGF binding to cell membrane EGFR triggers EGFR conformational change to allow homodimerization with another EGFR or heterodimerization with another ErbB family and then induces receptor activation followed by subsequent receptor internalization to the early endosome. In the early endosome, EGFR is either recycled back to the cell surface or sorted to the late endosome/lysosome for degradation in termination of signal transduction [26–28]. Several studies have shown that EGFR is capable of recruiting signaling molecules and transmit signals from endosome [29–32], however, a recent study reports that EGFR signaling takes place primarily at the plasma membrane and the function of EGFR internalization is to terminate signals induced by EGF binding to plasma membrane EGFR [33]. In this study, we demonstrate that at 1 min upon EGF binding GALNT1 knockdown decreased EGFR autophosophorylation, however, no significant effects of GALNT1 knockdown on EGFR dimerization were observed (data not shown). These results suggest that GALNT1 modulates EGFR O-glycosylation that confers EGFR conformation to a less active form rather than affecting the receptor dimerization, thereby regulates EGFR signaling at the phosphorylation level. We further found that, at a later time point, GALNT1 knockdown facilitated EGFR internalization and enhanced receptor degradation that is independent of the ubiquitylation pathway (data not shown) with an overall decrease in the protein levels of EGFR. These studies indicate that correct O-glycosylation is required in regulating the total EGFR protein levels on plasma membrane and its subsequent signal transduction. Therefore, we suggest that GALNT1 regulates EGFR phosphorylation and protein levels by modulating EGFR O-glycosylation and in turn regulates malignant behaviors of HCC cells.

We have previously reported that GALNT1 and GALNT2 are the two major polypeptide GalNAc-transferases in liver and HCC tissues; and overexpression of GALNT2 suppresses EGF-induced EGFR activation and enhances EGFR degradation [12]. In contrast, here we report that knockdown of GALNT1 suppresses EGF-induced EGFR phosphorylation, accelerates EGFR internalization, and enhances EGFR degradation. Although both GALNT1 and GALNT2 are polypeptide GalNAc-transferases, yet they have overlapping but distinct peptide substrate specificities [8]. In support of this finding, both GALNT1 and GALNT2 can glycosylate EA2 peptide, but only GALNT2 can glycosylate Apo C-III peptide [34]. Using the NetOGlyc4.0 O-glycosite prediction tool, we found 17 potential O-glycosites on the extracellular domain of EGFR. It is likely that GALNT1 and GALNT2 glycosylate different EGFR O-glycosites. Hence, the effects of GALNT1 and GALNT2 on EGFR glyco-phenotypes and EGFR properties, such as phosphorylation and degradation, manifest differently. Thus it is of great interest to further investigate the roles of GALNT1 and GALNT2 on each O-glycosite and the effects of each O-glycosylation on the properties of EGFR.

Cancer development and progression is a concerted effect of multifaceted contribution. Growth factors may be individually or simultaneously present in the microenvironment of tumors to exert their actions on cancer malignant behaviors. We demonstrate that GALNT1 overexpression enhances whereas knockdown suppresses HCC cell migration and invasion under the effects of 10% FBS. To unveil the underlying mechanism, the effects of several growth factors on HCC cell migration and invasion were analyzed. We found that knockdown of GALNT1 most significantly suppressed EGF-induced migration and invasion of HCC cells. However, in addition to EGF, knockdown of GALNT1 also significantly suppressed PDGF-induced invasion in HA22T cells and VEGF-induced invasion in HA22T and PLC5 cells. These results suggest that GALNT1 regulates HCC cell malignant behaviors most explicitly through the EGF pathway, but also through other growth factor signaling pathways. Indeed, in consistent with our findings *in vitro*, global gene expression analysis reveals that many functionally related genes associated with cell signaling, cell adhesion and motility, and protein transport and recycling were differentially expressed upon *GALNT1* knockdown in HA22T and PLC5 cells. *GALNT1* knockdown also resulted in differential expression of genes functionally related to development and differentiation. It is worthy to further study the effects of GALNT1 on cellular development and differentiation.

In summary, GALNT1 is frequently up-regulated in HCC compared with normal liver and higher *GALNT1* expression level is associated with poor five-year patient survival. Overexpression of GALNT1 enhances HCC cell malignant phenotypes but knockdown of GALNT1 suppresses HCC cell migration and invasion, and inhibits tumor metastasis in NOD/SCID mouse model. Furthermore, GALNT1 knockdown reduces O-glycan expressions on EGFR, decreases EGFR activation, and enhances EGFR degradation. These findings suggest that GALNT1 is a regulator of HCC malignant behaviors and may be a promising target of therapeutic drug development in the treatment and management of HCC.

## MATERIALS AND METHODS

### Human tissue samples

Postsurgical fresh HCC and adjacent non-tumor (paired) tissue samples were collected from patients receiving treatment at the National Taiwan University Hospital (NTUH). Samples to be used for protein extraction were stored at –80°C and for RNA extraction samples were preserved in RNA*later* (Qiagen Corp.) at 4°C overnight prior to storage at –80°C until use. For immunohistochemical analysis, samples were fixed with 4% (w/v) paraformaldehyde/PBS and embedded in paraffin until use. All tissues were collected under the consents of each patient prior to collection and this study was conducted under the approval of NTUH Ethics Committee.

### Cell lines and cell culture

Human hepatocellular carcinoma cell lines skHep1, HepG2 and Huh7 were purchased from Bioresource Collection and Research Center (Hsinchu, Taiwan) in 2008. Hep 3B, HA22T, and PLC5 were kindly provided by Dr. KF Chen (Department of Medical Research, NTUH) in 2013. All cell lines were authenticated by the provider on the basis of morphology, antigen expression, growth, DNA profile, and cytogenetics. Cell cultures were maintained with Dulbecco's modified Eagle Medium (DMEM) containing 10% FBS at 37°C under 5% CO_2_ atmosphere.

### Reagents and antibodies

Biotin-conjugated *Vicia villosa* agglutinin lectin (VVA) was purchased from Vector Laboratories. Actin beta and recombinant epidermal growth factor (EGF), fibroblast growth factor (FGF), hepatocyte growth factor (HGF), and insulin growth factor-I (IGF-1) and were obtained from Sigma. Platelet derived growth factor (PDGF) was purchased from PepproTech. Transforming growth factor beta (TGFβ) and antibodies against epidermal growth factor receptor (EGFR), phosphorylated EGFR (Tyr1068), and EGFR (Alexa594) employed in immunofluorescence were obtained from Cell Signaling Technology, Inc. Antibodies against EEA1 employed in immunofluorescence and EGFR in immunoprecipitation (IP) were obtained from Santa Cruz Biotechnology, Inc. Antibody against LAMP1 was purchased from BD Biosciences. Rabbit anti-human GALNT1 antibody was produced in-house. Briefly, human partial *GALNT1* (NM_020474) gene from HEK293 cells was obtained by RT-PCR using sense primer 5′-CGGGATCCTGCAACAAATGTGATGAAAA-3′ and antisense primer 5′-GGAATTCTTGTTCCATTCGAATT ACAT-3′. PCR products were cloned into pET30a (Novagen, Madison, WI) in generating GALNT1 recombinant proteins in BL21 *Escherichia coli*. Anti-human GALNT1 polyclonal antibody was raised by immunizing rabbits with GALNT1 recombinant proteins. The anti-serum was subjected to PVDF membrane for purification of anti-human GALNT1 antibody.

### cDNA synthesis and real-time RT-PCR

Total RNA from paired HCC tissues was extracted with TRIzol (Invitrogen) according to the manufacturer's protocol. Two micrograms of total RNA were transcribed into cDNA using Superscript III First-Strand cDNA Synthesis Kit (Invitrogen). *GALNT1* was quantified by real-time RT-PCR as described previously [9] using PCR System Mx3000P (Stratagene) with sense primer 5′-ATGGCCCAGTTACAATGCTC-3′ and anti-sense primer 5′-ATATTTCTGGCAGGGTGACG-3′ designed by Primer3 (v.0.4.0) algorithm. The relative quantity of gene expression was analyzed with MxPro Software (Stratagene) normalized to *GAPDH* expression.

### Immunohistochemistry

Paraffin-embedded HCC paired tissues were stained with anti-GALNT1 antibody, detected by Super Sensitive Link-label Immunohistochemistry Detection System (BioGenex), and visualized with 3,3-diaminobenzidine liquid substrate system (Sigma). Tissue sections were counterstained with haematoxylin for staining of nucleus. Negative control was done by replacing primary antibody with control IgG.

### Plasmid construction

Full-length human *GALNT1* from non-tumorous liver was cloned by using RT-PCR with sense primer 5′-CCCAAGCTTGGGCCATGAGA AAATTTGCATA-3′ and antisense primer 5′-CGGGATCCCTCAGA ATATTTCTGGCAGGG-3′. The RT-PCR products were cloned into pcDNA3.1A (Invitrogen, Life Technologies) to generate pcDNA3.1A/*GALNT1* gene and confirmed by DNA sequencing.

### Transfection

Overexpression of *GALNT1* gene was achieved by transfection of vector/pcDNA3.1A or *GALNT1/*pcDNA3.1A plasmids using Lipofectamine 2000 (Invitrogen, Life Technologies) according to the manufacturer's protocol. Cells stably expressing GALNT1 were selected and maintained with 400 μg/ml of G418. Stable knockdown of *GALNT1* gene was achieved by transfection of vector/pLKO (mock) or *GALNT1*/pLKO short hairpin RNA (shRNA) purchased from the National RNAi Core Facility of Academia Sinica (Taiwan) with Lipofectamine 2000 and selected and maintained with 2 μg/ml puromycin. Knockdown of GALNT1 with small interfering (si) RNA was achieved using 10 nM of SMARTpool siRNA oligonucleotides against *GALNT1* (Dharmacon Reseasrch, Thermoscientific) with RNAiMAX (Invitrogen, Life Technologies). Non-targeting siRNA was used as control.

### *In vivo* metastasis assay

Female NOD/SCID mice (4-week old) (National Laboratory Animal Center, Taiwan) were tail vein injected with 5 × 10^6^ of control (*n* = 6) or GALNT1 (*n* = 6) stable transfectants and were sacrificed after 60 days of injection for analysis of metastatic nodules. The animal study was reviewed and approved by the Institutional Animal Care and Use Committee (IACUC) of National Taiwan University College of Medicine.

### Transwell migration and matrigel invasion assays

Cells (3 × 10^4^) in serum free DMEM were loaded into the upper chamber and serum free DMEM (SF) with or without 10% FBS or EGF, FGF, HGF, IGF-1, PDGF, TGFβ, and VEGF were loaded to the lower chamber of the transwell migration or BioCoat Matrigel Invasion Chamber (Beckton Dickinson) systems. Cells were serum starved overnight prior to seeding and after 16 hours of migration and invasion cells were fixed and stained with 0.5% (w/v) crystal violet (Sigma). The number of migrated or invaded cells from 5 random fields was counted under the microscope. Results obtained were analyzed by student's *t*-test and graphed as mean ± SD.

### Internalization of EGFR and immunofluorescence microscopy

Cells were seeded on chamber slides and serum starved overnight prior to EGF 10 ng/ml stimulation for 0, 3, 5, 10, 15, and 30 min at 37°C under 5% CO_2_ atmosphere. After rinsing with PBS and fixation with 4% paraformaldehyde cells were immunostained with primary antibodies EGFR Alexa594, EEA1, and LAMP1, followed by FITC-conjugated goat anti-rabbit IgG as secondary antibody and counterstaining of nucleus with DAPI (Sigma).

### Immunoprecipitation and western blotting

Total cell lysates from cells pre-treated with or without benzyl-α-GalNAc were immunoprecipitated with protein G sepharose beads (GE Healthcare) conjugated with EGFR immunoprecipitation specific antibody, incubated at 4°C over night then subjected to neuraminidase enzyme digestion and visualized by Western blotting with biotin-conjugated VVA as described previously [12].

### Gene expression profiling and quantitative RT-PCR validation

Total RNA from control and GALNT1 siRNA knockdown HA22T and PLC5 cells was extracted in triplicates using GeneJET RNA Purification kit (Thermo Scientific) following the manufacturer's protocol and quantified by NanoDrop spectrophotometer (Bio-Rad). The RNA quality was monitored with Agilent 2100 Bioanalyzer (Agilent Technologies, Santa Clara, CA). cDNA prepared from 10 μg of total RNA was labeled with aa-dUTP using Invitrogen SuperScriptTM Plus Indirect cDNA Labeling System according to the manufacturer's protocol, followed by aa-cDNA column purification (QIAGEN, Valencia, CA). Alexa/CyDye was incorporated to aa-cDNA followed by column purification with Alexa/CyDye-cDNA cRNA purification (Qiagen). DNA yields were confirmed by 1% DNA agarose gel and visualized with Fuji image reader at 600V PMT. Agilent Gene Expression Hybridization Kit was used for hybridization according to the manufacturer's instruction. Briefly, 16 ul of dye labeled cDNA were hybridized to Agilent SurePrint G3 Human Gene Expression 8x60K v2 Microarray (G4851B). The microarrays were scanned on the Agilent DNA Microarray Scanner (US9230696) using one color scan setting for 8x60k array slides. The scanned images were analyzed with Feature Extraction Software 10.5.1.1 (Agilent). Features flagged in Feature Extraction as Feature Non-uniform outliers were excluded. Quantitative RT-PCR of selected genes expression was performed for validation as described above. The primers were designed with Primer3 (v.0.4.0) algorithm with the sequences freely available from the Entrez Nucleotide database. The list of primers is provided in Supplementary Table S3. All microarray experiments were performed in triplicates where three hybridizations were conducted for each *GALNT1* knockdown cells against the corresponding control. The microarray data were deposited in the GEO database, accession number GSE64628.

### Functional enrichment and network analyzes

The differentially expressed genes (fold-change > 1.5 and *p* < 0.05) were annotated based on gene sets. Gene sets are defined as groups of genes with related functions and derived from Gene Ontology. We used Fisher's exact test to assess if a gene set is enriched in differentially expressed genes. To simply interpret the results, enriched gene sets (*p* < 0.05) were organized graphically into a network, where a node and an edge represent each gene set and gene overlap between sets, respectively [35]. The gene overlap was scored by the arithmetic average of Jaccard coefficient $\text{JC}=\frac{\left|A\cap B\right|}{\left|A\cup B\right|}$ and Simpson coefficient $\text{SC}=\frac{\left|A\cap B\right|}{\min\left(\left|A\right|,\left|B\right|\right)}$ in which *A* and *B* are two gene-sets. An edge with an overlap score passing a threshold was presented in the networks. The networks were visualized by Cytoscape [36].

### Statistical analysis

Student *t*-test, paired *t*-test, Pearson's correlation for paired HCC tissues, and 2-way ANOVA were used for statistical analysis. Data are presented as means ± SD and *p* < 0.05 was considered statistically significant.

## SUPPLEMENTARY FIGURES AND TABLES




