# Supplementary material for: Knockdown of GALNT1 suppresses malignant phenotype of hepatocellular carcinoma by suppressing EGFR signaling
Source: Oncotarget. 2015 Feb 4;6(8):5650–65. doi: 10.18632/oncotarget.3117 (PMC4467392; doi:10.18632/oncotarget.3117)
Supplement: Supplementary file 1 [file oncotarget-06-5650-s001.pdf]

# SUPPLEMENTARY FIGURES AND TABLES

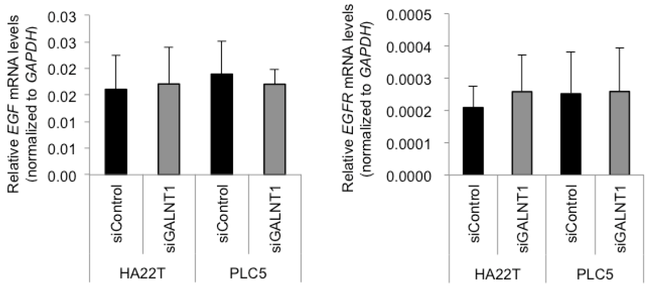


**Supplementary Figure S1: *EGF* and *EGFR* mRNA expression levels upon *GALNT1* knockdown.** No significant difference in the *EGF* and *EGFR* mRNA expression levels in *GALNT1* knockdown HA22T and PLC5 cells compared with control. Results were obtained from three independent *GALNT1* knockdown transfectants and their corresponding control and are represented as means ± SD.

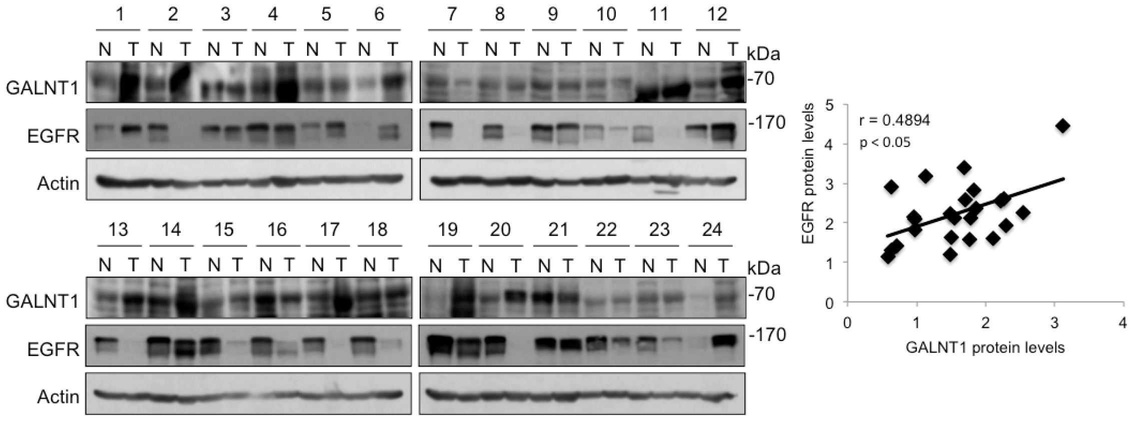


**Supplementary Figure S2: GALNT1 expression is correlated with EGFR expressions in HCC tumors.** Western blot of 24 paired HCC tissues displaying GALNT1 and EGFR expression levels (left panel). Pearson's correlation analysis indicates GALNT1 expression levels are moderately correlated with EGFR expression levels in HCC tumors ($r = 0.4894$, $p < 0.05$) (right panel).

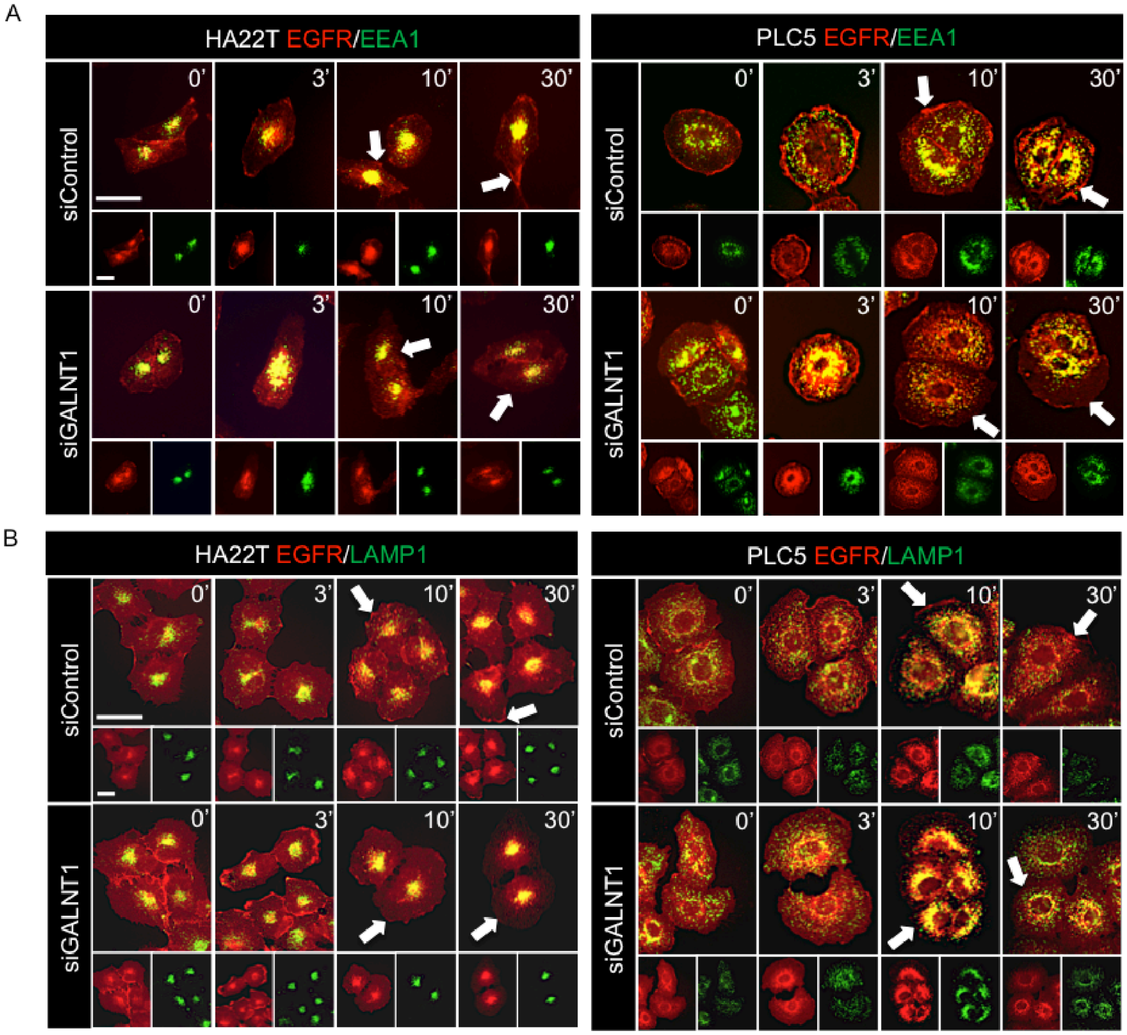


**Supplementary Figure S3: GALNT1 knockdown enhances EGFR internalization and enhances reduction of total EGFR. (A)** Immunofluorescence staining of EGFR (red), EEA1 (green) and co-localization (yellow) in HA22T (left panel) and PLC5 (right panel) cells. **(B)** Immunofluorescence staining of EGFR (red), LAMP1 (green) and co-localization (yellow) in HA22T (left panel) and PLC5 (right panel) cells. Cells were serum starved overnight and stimulated with EGF 10 ng/ml at 0, 3, 10, and 30 min. Staining of cell surface EGFR diminishes over time (arrow) in GALNT1 knockdown cells compared with control most evident at 10 min after EGF stimulation and with a persistent decrease in total EGFR at 30 min in GALNT1 knockdown cells compared with control.

**Supplementary Table S1: HCC patient information**

| Variable | No. of patients ($n$ = 151) | Percentage (%) |
|---|---|---|
| Age | | |
| Mean | 57.9 | |
| Max | 89 | |
| Min | 24 | |
| Gender | | |
| Male | 116 | 76.8 |
| Female | 35 | 23.2 |
| Clinical grade | | |
| I | 20 | 13.2 |
| II | 128 | 84.8 |
| III | 2 | 1.3 |
| IV | 1 | 0.7 |
| HBV | 114 | 75.5 |
| HCV | 44 | 29.1 |
| Distant metastasis | 17 | 11.3 |
| Vascular invasion | 50 | 33.1 |
| Survival months | | |
| Mean | 43.5 | |
| Max | 83.7 | |
| Min | 0 | |

**Supplementary Table S2: Differential expression of selected genes in GALNT1 knockdown HA22T and PLC5 cells**

**Supplementary Table S3: Real-time RT-PCR primer sequences**

| Gene name | Sense primer | Antisense primer |
|---|---|---|
| *SPTBN4* | AGCCTGTACTGTGTGCTTAG | AGACGAGATGTGTCAGGTCC |
| *KIF26B* | GAGGCGACCAAACAGTATCT | CTCCTTCCACGGTGTTTGAC |
| *WNT3A* | GACCTGGTCTACTACGAGGC | TGCCTTCAGGTAGGAGTTCT |
| *GDI2* | CTACGGAGGAGAGAGTGCAT | ACAAAGCTCCCTTCAGTCAC |
| *TMSB10* | AACCAGACATGGGGGAAATC | TGCTCAATGGTCTCTTTGGT |
| *FGF8* | GGTGTCTCCCAACAGGTAAC | AGCACAATCTCCGTGAAGAC |
| *TBCK* | CGCTGAAATGGGAGCCTTTA | ACGAGGGATACCTCACAGG |
| *EMP2* | GCGGCCTCCATTTATACAGA | TGCTGGATTTTTGCTTGTGTC |
| *MDM2* | CTTCGGAACAAGAGACCCTG | GAGTCCGATGATTCCTGCTG |
| *RAB22A* | GTATTGTGTGGCGGTTTGTG | TACATTGGTGCTAAGGCACG |
| *RHOQ* | TGGGTACCGGAACTTAAGGA | TTTCCTTGGCCACTGAAGAT |
| *ITSN1* | TCACTATCCAGCCAGGAGAC | GCTCTGAAGAGGTTACTGCC |
| *BCAR1* | ACAGCAAGTTCGTCATCCTC | CGACTCTTGACATGGGAGC |
| *FGD5* | GTGGAGATACGAGAGAGGCT | CTTACACCGGCTGAGATAGC |
| *SMAP1* | CAGCACCAGCTCATCCTATC | CTGTTCTGCTGTCCATTGGT |
| *SPTBN1* | GATCGACAAGTGGGAAGACC | TGGTAGGTTTGAGAGCGGTA |
| *GATA3* | GAAGGCAGGGAGTGTGTGAA | AGCTTGTAGTAGAGCCCACA |
| *HDAC5* | GTTGAGATCACAGGTGCCG | GACGTGTAGAGGCTGAACTG |
| *TET1* | CGAAGCACCTCTCTTAGCAG | TGTCTTGCATTGGAACCGAA |
| *LRP5* | CCCCTCAATTTCATAGCCCC | GTTCAGGATCGGCGGGTA |
| *MYADM* | CTCTCGGCCTCCATCATCTA | TAGAGGGGCCAGAGAACAAG |
| *GRM5* | CCAGAATGAGAAGAGCAGCC | GTGGACAGCATCATGGAGTT |
| *VAV1* | ATAACGTCGAGGTCAAGCAC | GCCTGCTGATGGTTCTCTTT |
| *DENND2A* | TGGTGTCTGAAGCCTTTGTC | GGAAGTTTTCCCTTGAGGCT |
| *IAPP* | GCAACAACTTTGGTGCCATT | GGGCAAGTAATTCAGTGGCT |
| *EGF* | CTGGACCCACCACACCAAAT | GTGCAATCACACCAAGAGGG |
| *EGFR* | TAACAAGCTCACGCAGTTGG | GTTGAGGGCAATGAGGACAT |
| *GAPDH* | ACAGTCAGCCGCATCTTCTT | GACAAGCTTCCCGTTCTCAG |
